# Twenty-five year trend in antipsychotic medication prescribing in England: challenges and opportunities

**DOI:** 10.1192/bjo.2025.10073

**Published:** 2025-07-16

**Authors:** Hannah Newman, David Branford, Richard Laugharne, Richard Byng, Rohit Shankar

**Affiliations:** 1 Peninsula School of Medicine, University of Plymouth, Plymouth, UK; 2 Livewell Southwest, Plymouth, UK; 3 Cornwall Partnership NHS Foundation Trust, Truro, UK

**Keywords:** Antipsychotic, psychotic disorders, prescribing, primary care, community

## Abstract

**Background:**

Antipsychotics are primarily indicated for psychotic disorders. There is increasing concern regarding their potential overuse for other conditions.

**Aims:**

To examine the change in the number of community prescriptions and corresponding costs for antipsychotics per head of population over 25 years (1998–2022) in England.

**Method:**

The data for 1998–2022 were obtained from two separate resources from the OpenPrescribing database: from 1998 to 2016 from their long-term trends data-set; and for 2017–2022 from the monthly medication prescribing data. The relevant British National Formulary subcategories 4.2.1 ‘antipsychotic drugs’ and 4.2.2 ‘antipsychotic depot injections’ were selected. The annual differences in prescriptions and the mean average annual increase were calculated. Scatter plots to visualise the yearly trend and Spearman testing to assess the strength of the correlations were done. The total annual costs of these medications were calculated for this time period.

**Results:**

The annual mean increase in the number of prescriptions was 287 548 in raw numbers and 4.27 per 1000 population. There is a statistically significant and strong positive relationship between time and the prescriptions of antipsychotics per 1000 population (Spearman correlation coefficient 0.995, *P* ≤ 0.001). This increasing trend is driven by the increase in oral antipsychotic drug prescriptions over time (Spearman correlation coefficient 0.995, *P* ≤ 0.001). Antipsychotic drug costs increased until 2011, reduced until 2016 and rose again during 2020–2022.

**Conclusions:**

This analysis suggests a worrying increasing trend in antipsychotic medication prescribing. Potential causal factors include off-licence use. Clinical practice and research implications are discussed.

Antipsychotic medications are licensed for the treatment of psychosis, for which they can be very beneficial. Psychosis is not a diagnosis, but a group of symptoms existing in many different diagnoses. These diagnoses include schizophrenia, bipolar disorder and severe depressive illness with psychotic symptoms.^1^ Psychotic symptoms, including hallucinations and paranoid delusions, can also occur in acute and chronic organic conditions such as delirium and dementias, and alongside other mental health disorders such as emotionally unstable personality disorder (EUPD). However, apart from post-traumatic stress disorder (PTSD), where antipsychotics are recommended as second line, the presence of psychotic symptoms is not an indication.

Antipsychotic medications carry the risk of significant unwanted effects such as weight gain, metabolic syndrome, cardiotoxicity and extra-pyramidal side-effects.^2^ Concerns have been raised that they may be frequently prescribed for off-label reasons, where the benefits may not outweigh the risks.^3^

An exploration of the English Prescription Cost Analysis data from 1998 to 2010 found an increase in the community prescriptions of drugs used for mental health disorders.^4^ Antipsychotic medication use increased by 5.1% per year on average. It concluded that this may be partly explained by longer-term treatment and an increase in the diagnosed population, but noted the low-dose prescribing of some antipsychotics.^4^ It was proposed that this would be consistent with other evidence indicating their use outside of severe mental illness.^4^

An analysis that focused on children and adolescents and utilised the English primary care Clinical Practice Research Datalink Aurum database found an increase in antipsychotic drug prescriptions over a 20-year period. The most likely indications for the prescriptions included psychosis, but also non-psychotic conditions such as autism spectrum disorder, anxiety disorders and conduct disorders.^5^

Another UK primary care study found that antipsychotic drug prescribing for people with personality disorders had increased between 2000 and 2016.^6^ The study suggested possible non-psychotic symptoms being treated with antipsychotics, i.e. impulsivity, aggression, poor interpersonal relationships and non-psychotic perceptual disturbances. It found greater frequency of prescribing in women, elderly patients and areas of greater deprivation. It also found a greater frequency in those with a history of adverse childhood events, who would likely benefit more from psychological therapies rather than long-term medication.

The NHS Business Services Authority studied, at a primary care level, the number of patients prescribed antipsychotic medications as well as the number of prescription items in England and found that both have increased over time from 2015–2016 to 2022–2023, consistent with the findings of this analysis.^7^ The study also included British National Formulary (BNF) guidelines section 4.2.3 ‘drugs for mania and hypomania’ under the umbrella term ‘antipsychotics’. The report found that the 1.30% increase in antipsychotic drug items prescribed from 2021–2022 to 2022–2023 was driven by an increase of a total 1.58% in the BNF section for oral antipsychotic drugs (4.2.1). The other two BNF categories showed decreases in prescriptions. Antipsychotic depot injection prescriptions decreased by 3.79%, and drugs used for mania and hypomania decreased by 0.68%. The NHS Business Services Authority analysis also noted that more females than males received at least one antipsychotic medication, although this was not as large of a difference as in other categories of mental health medications. It demonstrated a greater antipsychotic drug prescribing frequency in females in more deprived areas. Their work is not restricted to antipsychotics. The same report found that antidepressants had the largest number of prescription items, and that this increased by 2% from 2021–2022 to 2022–2023. The only BNF category found to decrease in that time period was hypnotics and anxiolytics, which had a reduction in prescription items of 2%.

The latest Adult Psychiatric Morbidity Survey (APMS) from NHS Digital estimates the prevalence of psychotic disorders to have remained broadly stable for several decades, increasing only slightly from 0.4 to 0.7% from 2007 to 2014.^8^ Another study found a slight increase in the prevalence of psychotic symptoms in a non-clinical population from 2000 to 2014, but this increase is disproportionate to the increase in antipsychotic drug prescribing.^9^

Given the stable prevalence of the licensed primary indication for use, i.e. psychosis and psychosis-related disorders, it would be expected that the number of antipsychotic drug prescriptions would not change significantly over time if population growth were controlled for. Although there are prevalence studies of antipsychotic drug prescribing in England covering a range of a few years, there are no longitudinal studies that have looked at the past 25 years of prescribing patterns.

This paper presents an analysis of data from the English OpenPrescribing database, to investigate the trend in community antipsychotic drug prescribing in England over 25 years.

## Aims

This analysis sought to answer the question, how has the number of prescriptions for antipsychotic medications and the total cost of antipsychotic medications prescribed in the community changed over the 25 years between 1998 and 2022 in England?

## Method

The OpenPrescribing database compiles anonymised data on all community medication prescriptions dispensed in England each month. It also contains a long-term trends data-set on annual data for all medications dispensed in the community in England from 1998 to 2016. This gives information on the drug names and dosage, number of prescriptions, prescription quantity and cost.^10^ The data analysed were compiled from both these data-sets.

The long-term trends data-set contains the raw data and the same data normalised per 1000 population to take population growth into account.^10,11^ The data for 2017 to 2022 was compiled from the OpenPrescribing monthly data and normalised by the same method, using mid-year estimates of the population in England from the Office of National Statistics.^12^ The two relevant BNF subcategories 4.2.1 ‘antipsychotic drugs’ and 4.2.2 ‘antipsychotic depot injections’ were selected and the total numbers of prescriptions per year calculated in raw numbers and from the normalised per 1000 population data. Scatter plots were produced to visualise the trend in the number of prescription items (normalised per 1000 population) per year for both oral and depot antipsychotics together and separately. The annual differences in the total number of prescriptions per 1000 population and the mean average annual increase were calculated. Following investigations of the distribution of the data-set, the data were treated as non-normally distributed. Spearman correlation testing was utilised to assess the strength of the correlations.

The annual total costs of antipsychotic medications for the same time period were calculated. There were no data available for this between the end of the long-term trends data-set, in 2016, and the more recent monthly medical prescribing data, which starts at 2019.

### Ethics statement

We confirm that we have read the journal’s position on issues involved in ethical publication and affirm that this report is consistent with those guidelines.

## Results

**Fig. 1:**
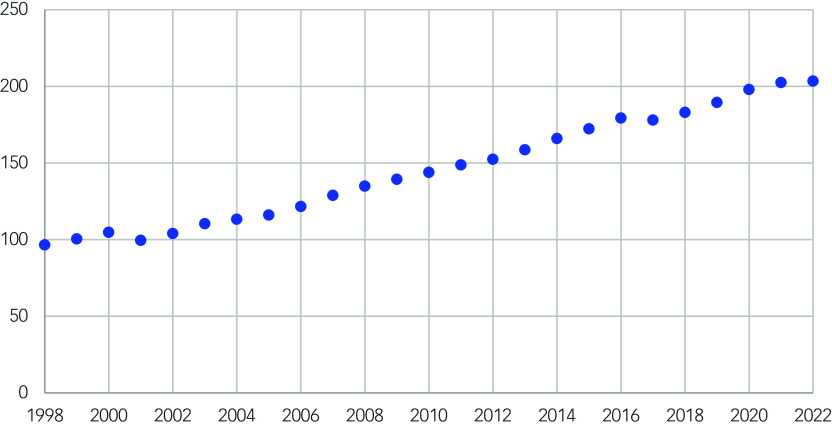
Scatter plot showing the number of prescriptions of antipsychotic medications per 1000 population over time (1998–2022).

There is a strong positive relationship between time and the number of prescriptions of antipsychotics per 1000 population that is statistically significant (Spearman correlation coefficient 0.995, *P* ≤ 0.001) (Fig. 1).

The range of the total number of prescriptions per 1000 people per year is 106.83 (minimum 96.56, maximum 203.39). The annual mean increase in the number of prescriptions is 287 548 in raw numbers and 4.27 per 1000 population (Table 1). All but two of the data points showed an increase from the previous year.

**Table 1:** Annual differences in total number of prescriptions of antipsychotic medications (raw and per 1000 population) and mean averages

Year	Number of prescription items	Annual difference	Number of prescription items (per 1000 population)	Annual difference (per 1000 population)
1998	4 714 300		96.56	
1999	4 924 800	210 500	100.44	3.88
2000	5 158 447	233 647	104.78	4.34
2001	4 922 071	−236 376	99.54	−5.24
2002	5 166 148	244 077	103.99	4.45
2003	5 512 428	346 280	110.41	6.42
2004	5 686 916	174 488	113.3	2.89
2005	5 872 073	185 157	116.04	2.74
2006	6 195 397	323 324	121.56	5.52
2007	6 618 676	423 279	128.82	7.26
2008	6 988 387	369 711	134.87	6.05
2009	7 272 332	283 945	139.33	4.46
2010	7 575 491	303 159	143.9	4.57
2011	7 897 349	321 858	148.71	4.81
2012	8 151 385	254 036	152.38	3.67
2013	8 541 357	389 972	158.57	6.19
2014	9 013 048	471 691	165.94	7.37
2015	9 439 867	426 819	172.3	6.36
2016	9 911 775	471 908	179.34	7.04
2017	9 900 917	−10 858	178.01	−1.33
2018	10 244 900	343 983	183.02	5.01
2019	10 666 681	421 781	189.51	6.49
2020	11 192 094	525 413	197.92	8.41
2021	11 448 039	255 945	202.49	4.57
2022	11 615 445	167 406	203.39	0.9
**Mean**	7 785 213	287 548	145.80	4.27

**Fig. 2:**
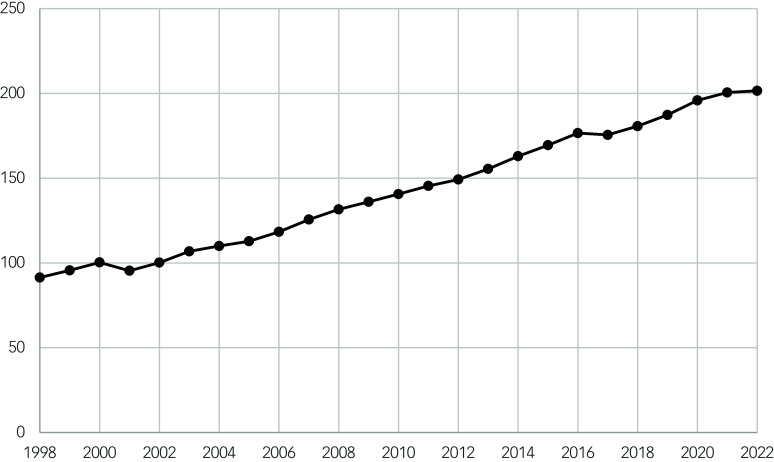
Scatter plot showing the number of prescription items for oral antipsychotics per 1000 population over time (1998–2022).

**Fig. 3:**
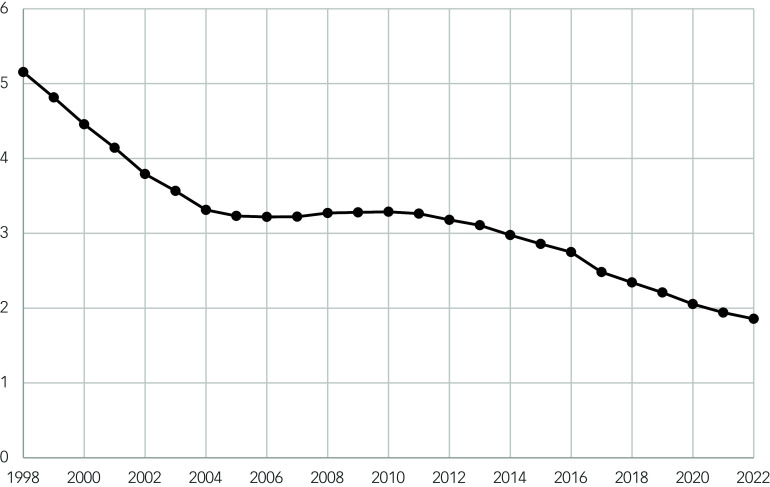
Scatter plot showing the number of prescription items for depot antipsychotics per 1000 population over time (1998–2022).

This increasing trend is driven by the increase in oral antipsychotic drug prescriptions over time (Spearman correlation coefficient 0.995, *P* ≤ 0.001) (Fig. 2). Contrastingly, depot antipsychotics showed a strong negative correlation between time and the number of prescriptions per 1000 population (Spearman correlation coefficient –0.964, *P* ≤ 0.001) (Table 2, Fig. 3). However, the size of the annual decrease is small, and does not mitigate the larger increase in oral antipsychotic drug prescriptions.

**Table 2:** Annual total oral and depot antipsychotic drug prescriptions per 1000 population

Year	Oral antipsychotics (per 1000 population)	Depot antipsychotics (per 1000 population)
1998	91.41	5.15
1999	95.62	4.82
2000	100.32	4.46
2001	95.39	4.14
2002	100.20	3.79
2003	106.85	3.57
2004	109.99	3.31
2005	112.80	3.23
2006	118.34	3.22
2007	125.59	3.22
2008	131.60	3.27
2009	136.05	3.28
2010	140.62	3.29
2011	145.44	3.26
2012	149.20	3.18
2013	155.46	3.11
2014	162.96	2.98
2015	169.45	2.86
2016	176.59	2.75
2017	175.53	2.48
2018	180.68	2.34
2019	187.30	2.21
2020	195.86	2.05
2021	200.55	1.94
2022	201.53	1.86

**Fig. 4:**
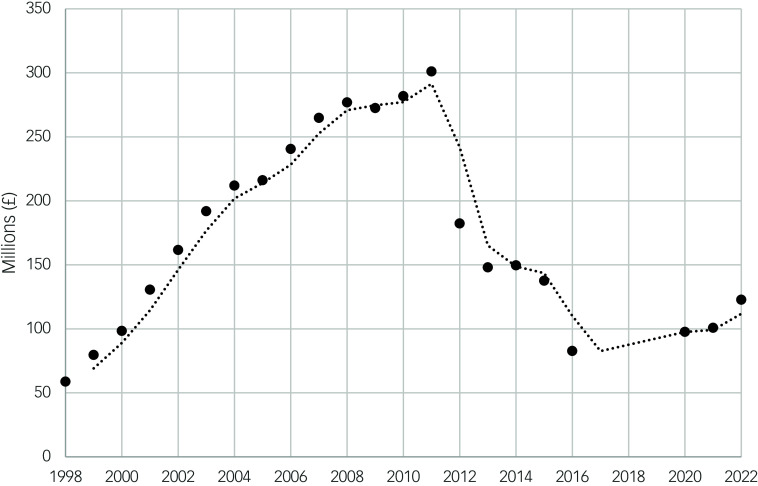
Scatter plot showing the total annual cost of antipsychotic medications over time (1998–2022) and moving average trend.

The cost of antipsychotic medications increased annually until 2011. The trend after 2011 is less clear: there was a reduction in the annual cost until 2016, and a smaller rise again between 2020 and 2022. However, there is a data gap from 2017 to 2019 (Table 3, Fig. 4).

**Table 3:** Annual total cost of antipsychotic medications

Year	Total cost (£)
1998	58 715 700
1999	79 555 900
2000	98 382 146
2001	130 593 760
2002	161 616 581
2003	191 835 994
2004	211 899 090
2005	216 043 818
2006	240 522 134
2007	264 748 645
2008	276 897 769
2009	272 485 140
2010	281 814 056
2011	301 107 813
2012	182 267 328
2013	148 005 588
2014	149 595 688
2015	137 521 300
2016	82 639 148
2017^a^	
2018	
2019	
2020	97 573 563
2021	100 752 492
2022	122 689 859

a .The data on antipsychotic costs between 2016 and 2020 were not available.

## Discussion

This analysis showed a significant increase in community antipsychotic drug prescriptions in England over 25 years, between 1998 and 2022, that is driven by an increase in oral antipsychotic medications. Conversely, a decrease in depot antipsychotic drug prescribing was found. This increase is despite taking population growth into account, and the stable estimated prevalence of psychotic disorders in England, at least up until 2014.^8^

There was a less clear trend of the annual cost of antipsychotic medications: after a steady increase up to 2011, there was a reduction in the annual cost. This less likely reflects the ‘patent cliff’ phenomenon, whereby the patents of several commonly used antipsychotics have ended, allowing the use of cheaper generic forms.^13^ However, the total cost of these medications remains significant, at over £120 million in 2022, and the final few data points could suggest an increasing trend again. Further monitoring over the next few years would be needed to establish if this is the case.

The finding of an increase in community antipsychotic drug prescriptions is in keeping with the earlier exploration of the Prescription Costs Analysis data from 1998 to 2010^4^ and the observed trend in antipsychotic drug prescribing in children from 2000 to 2019,^5^ and the NHS Business Services Authority analysis.^7^ Potential mediators of this result could be a greater proportion of people with psychotic disorders receiving treatment, greater numbers of antipsychotic drug prescriptions per person with a psychotic disorder (antipsychotic polypharmacy) or an increase in the use of antipsychotics for other conditions.

### Polypharmacy

Antipsychotic monotherapy is generally recommended in guidelines over polypharmacy.^14,15^ However, polypharmacy is still reported to be common, and some researchers recommend a more individualised approach, suggesting that there is benefit of polypharmacy in some cases.^16–19^ An increasing trend in antipsychotic polypharmacy could explain the increasing trend in antipsychotic drug prescriptions. However, a systematic review concluded that the rate of antipsychotic polypharmacy in Europe had not significantly changed over time (from the 1970s to 2009),^20^ and a UK study of primary care data found that the annual antipsychotic polypharmacy prevalence between 2001 and 2013 fluctuated between 5 and 6%, i.e. did not suggest an increase in such prescribing.^21^

Although antipsychotics are the focus of this work, increasing prescribing can be seen in the majority of classes of psychotropics.^7^ Polypharmacy in general is increasing, but it is unclear if the polypharmacy is more than one antipsychotic medication for the same person or the general rise across psychotropics in general.

### Pharmaceutical and community context

The circumstances in the pharmaceutical and psychiatric fields over this period should be taken into consideration. The introduction of ‘atypical’ antipsychotics into clinical practice in 1990s and 2000s, which were touted to have higher efficacy and better tolerability, may have increased prescribing.^22,23^ However, as there has been little further development in this pharmaceutical area, this factor would be more likely to have produced a plateau effect.

The increasing political requirement for shared-care mental health agreements between secondary and primary care since 1997 may also have led to an increase in prescriptions from general practices.^24,25^ In the early 2000s, the ‘New Ways of Working for Psychiatrists’ UK initiative emphasised the role of primary care in supporting people with long-term mental health conditions, leading to a shift from secondary to primary care management, which could include psychotic disorders.^26^ This could feasibly increase the number of prescriptions of antipsychotics being prescribed by general practitioners (GPs) rather than psychiatrists, simply by GPs taking over the prescribing and dispensing. However, again it would not explain the continuous positive trend of increased prescriptions, as a plateauing effect would have been more likely after this change.

The gradual decrease in primary care depot antipsychotic drug prescriptions is an interesting observation. This could reflect an absolute reduction and perhaps there has been a reciprocal shift of more complex patients toward secondary care services, which are more likely to provide depot medications, and could explain this change.

### Off-licence use

As discussed, there are concerns that antipsychotic medications are being increasingly used for off-licence purposes. The above possible casual factors do not fully explain the increasing trend in antipsychotic drug prescribing when this is considered against the estimated stable prevalence of psychotic disorders.^27^ Our findings support the idea that this trend is likely, at least in part, to be attributable to increased prescribing for off-licence purposes.

It is possible that the prevalence of psychotic disorders may not truly be stable: there is significant variance between estimates and sample sizes tend to be small.^8^ Additionally, the results from the APMS between 2015 and 2022 are not yet available. Affective psychosis, such as bipolar disorder and psychotic depression, is included in the 2014 APMS estimate of psychotic disorders.^8^ Bipolar disorder is also considered separately in its own chapter, in which the overall prevalence is estimated to be 2%. This was not considered separately in the 2007 survey and the authors state that before the 2014 survey, there were no lifetime prevalence rates available for the UK general population, but that these findings are consistent with those from epidemiological studies in other countries.^8^ Antipsychotics are one type of medication that may be used as a mood stabiliser or for psychotic symptoms in bipolar disorders. Whether they are used in greater or lesser amounts than alternatives (for example, lithium, or some antiseizure medications) may also be influenced by trends in the prescribing of those alternatives. For example, sodium valproate has recently received more negative attention.^28–30^ This may have already had an impact on shifting the focus toward antipsychotic medications or may have the potential to do so in the near future.

Being used outside of indicated disorders does not inherently mean that the use is not warranted. Antipsychotics are also used, with some limited evidence to support, management of non-psychotic symptoms in challenging behaviour associated with autism spectrum disorder^31^ and agitation in EUPD.^6^ EUPD is no longer a diagnosis of exclusion and is more actively treated.^32^ Some individuals often have symptoms that are ‘on the edge’ of psychosis, bipolar disorder or PTSD. These symptoms might include emotional instability, where elation lasts days; arousal owing to threat, similar to PTSD; disabling paranoia with overvalued ideas and intense second person or internal voices. Although not evidenced indications, such symptoms are hard to bear for patients, and some GPs and psychiatrists prescribe antipsychotics off-licence for their management. This data-set did not allow for exploration of such patient demographics.

Other contributory groups could be children and young people and the elderly. The increasing trend of antipsychotic drug prescribing has been a concern in children and young people internationally, where they are more likely to be used for non-psychotic symptoms such as challenging behaviours.^33–35^ There have been efforts to reduce the use of antipsychotics in elderly people and specifically in people with dementia for over a decade.^33,36^ These findings could signify an inadequate campaign that requires further efforts and different strategies, in the general population and specifically in these groups.

This analysis and several others have focused on the more readily available community data, the majority of which is primary care data. Clinicians are in the difficult position of trying to provide their patients’ short-term relief from their pain and distress in an environment of social adversity, healthcare austerity and limited availability of alternatives such as psychological therapies. One such perceived solution may well be the off-licence use of antipsychotic medications. Patient expectations of pharmacological treatment solutions and lack of knowledge of side-effects, and reluctance to stop medications started by specialists may also contribute.^37,38^ Halting this trend will require collaboration between primary and secondary care services, and a shift in approach from medicalisation to addressing psychosocial causes of distress, i.e. treating and preventing the cause rather than just the symptoms. This is increasingly challenging in an increasingly austere environment for mental health services and patients.^6,37^

### Limitations

This data-set only includes community prescriptions in England and does not include data on in-patient or hospital prescribing. Thus, this data-set is likely to underestimate the true use of antipsychotics across all healthcare services. Prescriptions of antipsychotic medications that are usually prescribed by specialist services, such as clozapine and depot medications, may also not be captured. However, most patients under secondary psychiatric care will be prescribed their medication by primary care with advice from the specialist. Psychotic disorders tend to fall under the category of severe and enduring mental illness, and it has been estimated that only around 30% of patients with severe mental illness are managed only by primary care.^39^

However, a recent study found that most patients on antipsychotics were managed in primary care without psychiatry review. The study is complementary to this analysis: whereas our analysis focused on primary care prescribing in England over 25 years, this study focused on antipsychotic management in primary care in Wales, from 2011 to 2020. The study found that the prevalence of adults prescribed long-term antipsychotics increased, but that the proportion prescribed to patients with serious mental illnesses decreased. They also highlighted the need for cardiometabolic monitoring.^40^

Different regions also have different arrangements for shared care between the local primary and secondary services, which may mean that secondary care may be prescribing ongoing antipsychotic treatments. However, the evidence on this^38^ is over a decade old, when ‘New Ways of Working’ was still in its infancy. It is highly likely that significantly more prescribing is happening via primary care.

The data on antipsychotic costs between 2016 and 2020 were not available. It is interesting to note that before 2016, the cost trend was going downward, but started to rise again from 2020 onward. It would have helped to identify the inflection year. This could have given opportunities to better understand if the reductions in cost were because of direct prescribing influences or other external factors such as system changes in accounting. A possible explanation for a recognised downward trend in cost and upward trend in prescriptions (hypothesised in Figs 1 and 4) could be the knock-on impact of national campaigns around antipsychotic drug prescribing in special populations such as those for intellectual disability and dementia. This could have led to lower doses of antipsychotics, thus reducing costs, but not prescribing frequency. For example, in the intellectual disability population in England, 17% were on antipsychotics in 2015, which reduced to 15.9% over the following 5 years.^41^ However, the noted downward shift in antipsychotic drug prescribing in the second half of the decade was associated with a significant increase of prescribing of other psychotropic medication outside of licensed indication, particularly antidepressant and antiseizure medication.^42,43^ In addition, the possible gains made were lost because of the COVID-19 pandemic.^44^

The data-set does not include information on the demographics of the patients for whom the medications are prescribed, nor any information on the duration of the prescription items or the patient’s total dose. Each medication is listed separately according to the dosage. Therefore, a patient might be on multiple forms to add up to a total higher dose and this would be listed as separate prescription items. It is possible that shorter prescriptions were being issued, resulting in more prescriptions being required for the same individual and same medication; however, there does not appear to be a clinical impetus for such a change.

Patients may be prescribed different medications within the yearly period, and so would have multiple observations within that time point, and also across multiple years. Non independence of the observations violate one of the assumptions of the statistical test, and may inflate or deflate the correlation coefficient. Unfortunately, because of the database employed, it has not been possible to address this concern. However, the finding remains of an increasing trend of prescriptions, whether this be for multiple medications per individual, or more individuals receiving treatment, or a combination of both.

A strength is that all the community prescription items of England across the 25 years are included in the data-set, rather than a sample. Further, the limitations of lack of demographics and generalisability are somewhat mitigated when considered in context of the body of evidence as discussed above.

### Implications for clinical practice

This study suggests that there is a rising trend in the prescribing of antipsychotic medications in excess of the estimated stable prevalence of psychotic disorders. This could indicate an increase in use that may be less evidence-based. Given the potential of these medications for serious negative effects, this is concerning, and those prescribing these medications should be aware of this trend and consider if it may be at play in their own work settings. This is particularly relevant in more vulnerable populations of people with severe mental illness or intellectual disabilities, who have a greater risk of physical health problems and premature mortality,^45,46^ and in children and the elderly. It is recommended that people with psychotic disorders receive an annual physical health check, in part because of the physical side-effects of antipsychotic medications.^47^ It should be considered if these should also be carried out for people prescribed antipsychotic medications who do not have a psychotic disorder. Additionally, if unnecessary prescriptions were reduced, this could reduce the burden on primary care in providing these. Secondary care clinicians should ensure that they give adequate advice on discharging a patient on antipsychotic medication to primary care, regarding reviewing the medication.

### Implications for policy

There are currently a number of projects seeking to optimise and deprescribe antipsychotic medications, including in people with intellectual disabilities^48^ and schizophrenia.^49^ Given that there are guidelines regarding the indications for use of antipsychotic medications, it may be useful to address why policies may not be being adhered to, and the challenges faced by clinicians and patients in doing so. Enhanced prescribing audits, improved GP training or better integration with secondary care review processes can be used to strengthen our findings. Reducing prescriptions of antipsychotic medications would likely also have an economic impact, by reducing the amount spent by the healthcare system on this medication class.

### Implications for research

Considering the increase in prescribing of antipsychotics over time, it would be important to research the indications for the prescription. It is possible that an increase is for challenging behaviour in autism spectrum disorders and this needs to be examined. The OpenPrescribing database continues to compile data on community prescribing, and this could be of interest to establish the continuing trend. Analysis of this could help to determine the success of the above strategies and predict future trends. If antipsychotic drug prescribing were to decline, it would be of interest to see if prescriptions of other psychotropic medications were also affected. Any impact of recent changes in approach to sodium valproate could be explored.^50^ Further, a quality improvement methodology and having a research register might enable an outline on how medication optimisation can be delivered. Further work is needed to understand and address the increasing use of antipsychotic medications in different populations. Exploration of secondary care and in-patient prescribing, patient demographics and the indications given for antipsychotic drug prescriptions would help to illuminate this issue.

The absence of demographic information and similar missing information, as identified by our study, needs to be addressed by future studies. This might be achieved by examining methodologies for linking with other data-sets (such as Clinical Practice Research Datalink or Hospital Episode Statistics) and evaluating if gaps still remain, and addressing them accordingly.

It is important to note that although our discussion presents plausible explanations, such as shared-care models and off-label use, these are hypotheses rather than definitive conclusions.

## Data Availability

The data that support the findings of this study are available via: https://openprescribing.net. Further details of analysis are available from the corresponding author upon reasonable request.
